# The Multiple DSF-family QS Signals are Synthesized from Carbohydrate and Branched-chain Amino Acids via the FAS Elongation Cycle

**DOI:** 10.1038/srep13294

**Published:** 2015-08-20

**Authors:** Lian Zhou, Yonghong Yu, Xiping Chen, Abdelgader Abdeen Diab, Lifang Ruan, Jin He, Haihong Wang, Ya-Wen He

**Affiliations:** 1 State Key Laboratory of Microbial Metabolism, School of Life Sciences & Biotechnology, Shanghai Jiao Tong University, Shanghai 200240, China; 2 College of Life Sciences, South China Agricultural University, Guangzhou 510650, China; 3 State Key Laboratory of Agricultural Microbiology, College of Life Science and Technology, Huazhong Agricultural University, Wuhan, 430070, China

## Abstract

Members of the diffusible signal factor (DSF) family are a novel class of quorum sensing (QS) signals in diverse Gram-negative bacteria. Although previous studies have identified RpfF as a key enzyme for the biosynthesis of DSF family signals, many questions in their biosynthesis remain to be addressed. In this study with the phytopathogen *Xanthomonas campestris* pv. *campestris* (*Xcc)*, we show that *Xcc* produces four DSF-family signals (DSF, BDSF, CDSF and IDSF) during cell culture, and that IDSF is a new functional signal characterized as *cis*-10-methyl-2-dodecenoic acid. Using a range of defined media, we further demonstrate that *Xcc* mainly produces BDSF in the presence of carbohydrates; leucine and valine are the primary precursor for DSF biosynthesis; isoleucine is the primary precursor for IDSF biosynthesis. Furthermore, our biochemical analyses show that the key DSF synthase RpfF has both thioesterase and dehydratase activities, and uses 3-hydroxydedecanoyl-ACP as a substrate to produce BDSF. Finally, our results show that the classic fatty acid synthesis elongation cycle is required for the biosynthesis of DSF-family signals. Taken all together, these findings establish a general biosynthetic pathway for the DSF-family quorum sensing signals.

DSF (diffusible signal factor) represents a novel structural class of quorum sensing (QS) signals with the cis-2-unsaturated fatty acid moiety in diverse Gram-negative bacteria pathogens^1^,^2^,^3^. DSF was first characterized as *cis*-11-methyl-2-dodecenoic acid and found responsible for the regulation of extracellular, degradative enzyme and extracellular polysaccharide biosynthesis in *Xanthomonas campestris* pv. *campestris* (*Xcc*), the causal agent of black rot in crucifers^4^,^5^. In the last few years, DSF-like signals have been identified in a diverse range of bacterial species. For example, the rice bacterial blight pathogen *X. oryzae* pv. *oryzae* (*Xoo*) produces DSF, BDSF (*cis*-2-dodecenoic acid), and CDSF (*cis*-11-methyldodeca-2,5-dienoic acid)^6^. Although *Burkholderia* sp. mainly produce BDSF, some also produce a low level of DSF or CDSF^7^. *Stenotrophomonas maltophilia* strain WR-C was found to produce a range of extracellular fatty acids, including DSF and seven structural derivatives^8^. XfDSF produced by the xylem-limited plant pathogen *Xylella fastidiosa* has been recently characterized as tetradecenoic acid^9^. The human opportunistic pathogen *Pseudomonas aeruginosa* PAO1 produces *cis*-2-decenoic acid^10^. Recently, a DSF-like signal LeDSF3, which regulates the biosynthesis of the antifungal polycyclic tetramate macrolactam HSAF in *Lysobacter enzymogenes*, was determined to be 13-methyltetradecanoic acid^11^. These findings show that production of multiple DSF-like signals is a common attribute in various bacterial species; thus, the term “DSF family signals” was proposed to describe all the DSF-like signals^6^. It remains to be determined why and how bacteria produce multiple DSF-family signals.

*Xcc* has evolved a DSF-dependent QS system for genetic regulation at the community level. An early transposon mutagenesis analysis unveiled that a cluster of *Xcc* genes, designated *rpfABCDEFG* (for regulation of pathogenicity factors), are involved in the production of EPS and extracellular enzymes^12^. RpfC and RpfG constitute a two-component system to sense and to transduce the DSF signal^13^,^14^,^15^. RpfF is the key enzyme involved in the synthesis of DSF-family signals. A crystalline structure analysis of *Xcc* RpfF has unveiled a hydrophobic pocket composed of several hydrophobic residues, which is likely to be the putative DSF precursor docking site for DSF biosynthesis^16^. Bi *et al.*^17^ reported that Bcam0581, a *Burkholderia cepacia* protein showing 37% identity to RpfF of *Xcc*, displays both acyl-ACP thioesterase and dehydratase activity. Bcam0581 uses 3-hydroxydedecanoyl-ACP as substrate to produce BDSF. Whether RpfF has similar enzymatic activity remains to be investigated. Recently, RpfB in *Xcc* and *Xyllela fastidiosa* was found to be a fatty acyl-CoA ligase required for the degradation of long chain fatty acids^18^,^19^. These findings suggest that *Xcc* and *Xyllela fastidiosa* might have developed a fine-tuned system to control DSF-family signals biosynthesis and degradation. Characterization of the biosynthetic and degradation pathways will further our understanding of DSF-dependent quorum sensing and quenching mechanisms.

Fatty acids are precursors to a variety of important building blocks such as phospholipids, sterols, secondary metabolites and signaling molecules^20^. In bacteria, fatty acid synthesis (FAS) is catalyzed via a set of distinct monofunctional enzymes (type II)^21^,^22^. FAS utilizes acetyl coenzyme A (acetyl-CoA) as the primer and malonyl-CoA as the chain extender via a classic FAS elongation cycle. Four reactions (condensation, reduction, dehydration, and reduction) are required to complete each round of fatty acid elongation^22^,^23^. Typical for the fatty acid profiles of the genus *Xanthomonas* is the occurrence of many branched and hydroxyl-branched fatty acids^24^,^25^. Little is known of the biosynthetic mechanisms of these fatty acids except that *Xanthomonas oryzae* pv. *oryzae* FabD, FabB, FabH and FabV have been expressed and crystallized^26^,^27^,^28^,^29^.

Bacteria are capable of producing both straight chain and branched-chain fatty acids (BCFA). Some bacteria like *E. coli* produce predominantly straight-chain fatty acids whereas others like *Streptomyces* and *Staphylococcus* produce predominantly BCFAs^30^,^31^,^32^. BCFAs are synthesized from branched-chain acyl-CoA primers with malonyl-ACP as the chain extender^33^. Among the DSF-family signals produced, DSF and CDSF are branched-chain fatty acids. Bi *et al.*^17^ proposed that the 11-methyl substitution of DSF is probably derived from the branched-chain amino acid leucine. The metabolic origins of DSF-family signals require further investigation.

This study aimed at the thorough elucidation of the biosynthetic mechanism of DSF-family signals. We identified a novel functional IDSF and elucidate the metabolic origins of DSF, BDSF and IDSF. We further demonstrated that RpfF has both dehydratase and thioesterase activities, and that DSF-family signals are synthesized via the FAS elongation cycle. Thus, we establish a general biosynthetic pathway for DSF-family signals.

## Results

### *Xcc* produces four DSF-family signals during cell culture

**Figure 1:**
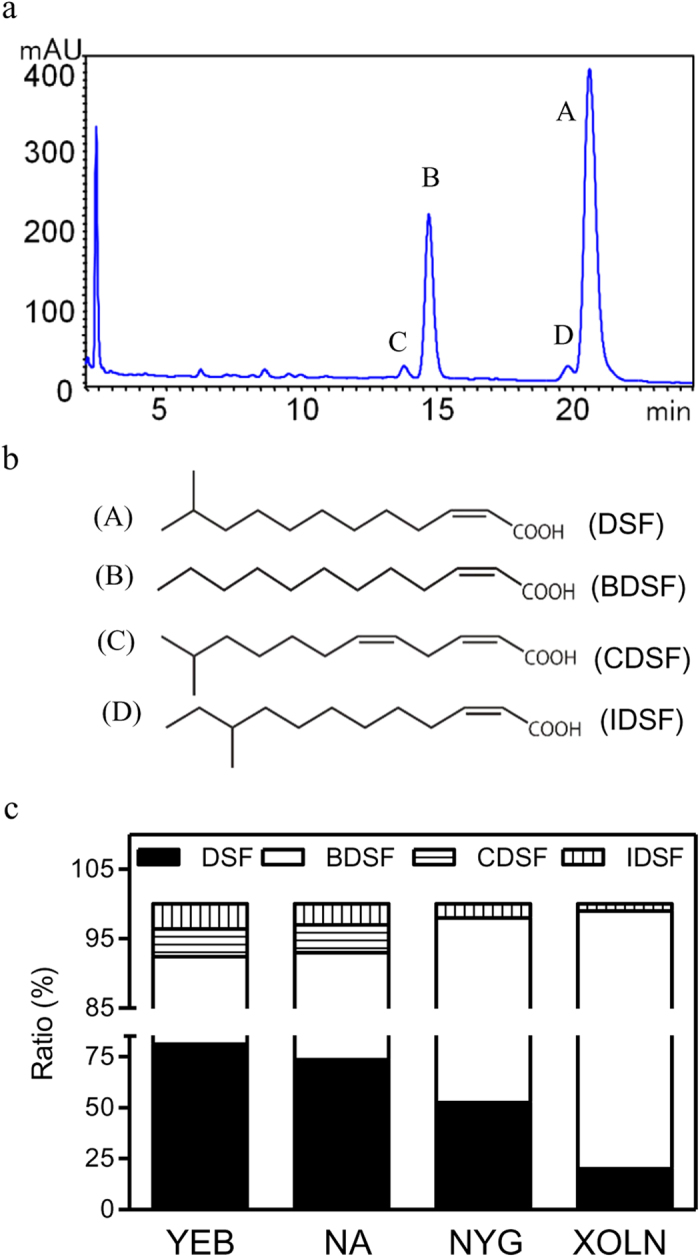
DSF-family signal production of *Xcc*. (**a**) HPLC analysis of the compounds in fractions A–D extracted from the strain Δ*rpfC* culture supernatant. (**b**) The chemical structures of DSF, BDSF, CDSF and IDSF as determined by liquid chromatography-mass spectrometry and/or nuclear magnetic resonance analysis. (**c**) The proportions of DSF, BDSF, CDSF and IDSF produced by strain Δ*rpfC* supernatant in media YEB, NA, NYG, and XOLN.

DSF-family signals produced by *Xcc* strain XC1 (*rpfC*) in NA medium were extracted as previously described^6^. Further separation with HPLC identified four active fractions with retention times of 14.3 (C), 15.2 (B), 20.3 (D), and 21.1 (A) min respectively, which showed UV absorption maxima at 212 nm and strong DSF activity (Fig. 1a and Supplementary Fig. S1c). Liquid chromatography-mass spectrometry (LC-MS) analysis confirmed that the three compounds in peaks A, B and C were the previously identified DSF-family signals DSF, BDSF and CDSF (Fig. 1b and Supplementary Fig. S1d–f). The spectrometry data of fraction D suggested a new member of DSF-family signals (IDSF), the characterization of which will be discussed in the following section.

DSF-family signal production was further studied in media YEB, NYG and XOLN. The results showed that the relative proportions of DSF, BDSF, CDSF and IDSF production at stationary growth phase varied from rich YEB medium (81.1%, 11.3%, 4.0%, and 3.6%, respectively), to NYG (52.5%, 45.5%, 0%, and 2.0%, respectively), to nutrient poor XOLN medium (20.0%, 79.0%, 0%, and 1.0%, respectively) (Fig. 1c). None of the four signals were detected in the supernatant extract of the *rpfF, rpfC* double deletion mutant in NA medium (Supplementary Fig. S1b), suggesting that they are synthesized by RpfF in *Xcc*.

### IDSF is a new functional DSF-family signal

IDSF had an HPLC retention time of 20.3 min, and strong DSF-like activity (Fig. 1a). LC-MS analysis showed that the m/z of IDSF is 212.1693, suggesting a molecular formula of C_13_H_24_O_2_ (Supplementary Fig. S2a). ^1^H NMR and ^13^C NMR spectral analysis revealed that IDSF is the novel unsaturated fatty acid *cis*-10-methyl-2-dodecenoic acid (Fig. 1b, Supplementary Figs S2 and 3), which is identical to DSF except for the methyl group present at the C10 position.

Exogenous addition of IDSF at 1.0 μM to a culture of the 8004 Δ*rpfF* strain could disperse aggregate formation (Supplementary Fig. S4a). To further compare the biological activities of the four DSF-family signals, DSF, BDSF, CDSF and IDSF were added to *rpfF* mutant-inoculated NA medium at three different final concentrations; 0.2 μM, 0.5 μM and 1.0 μM. At a final concentration of 0.5 μM, DSF and IDSF are more active than BDSF or CDSF with respect to EPS production and protease activity (Supplementary Fig. S4b,c). At both 0.5 and 1.0 μM, CDSF is less active than its three analogues (Supplementary Fig. S4b,c).

### *Xcc* mainly produces BDSF in the presence of carbohydrates

**Figure 2:**
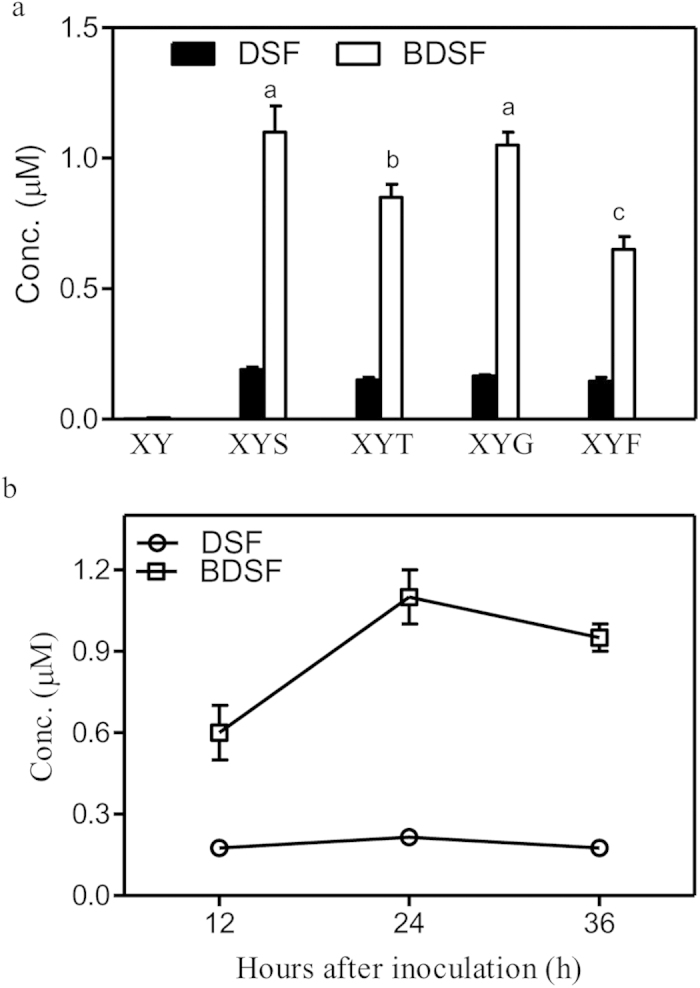
The effects of various carbohydrates on BDSF biosynthesis. (**a**) DSF and BDSF production of strain Δ*rpfC* in media XY, XYS, XYT, XYG and XYF. XY medium contains 0.7 g/L K_2_HPO_4_, 0.2 g/L KH_2_PO_4_, 1.0 g/L (NH_4_)_2_SO_4_, 0.1 g/L MgCl_2_, 0.01 g/L FeSO_4_, 0.001 g/L MnCl_2_, 0.2 g/L yeast extract, pH7.0. XYS medium: XY medium supplemented 2.0 g/L sucrose; XYT: XY medium supplemented with 2.0 g/L starch; XYG: XY medium supplemented with 2.0 g/L glucose; XYF: XY medium supplemented with 2 g/L fructose. (**b**) DSF and BDSF production time courses in XYS medium. Data are means ± one standard deviation of three independent assays. Different letters indicate significant differences between treatments (LSD at *P* = 0.05).

In order to understand how medium composition influences the production of DSF-family signals, a range of media were developed based on the salt composition of XOLN medium (Supplementary Table 2)^34^. Medium XY contains XOLN salts, 0.2 g/L yeast extract, and was used as a base medium for analyzing DSF-family signal production. In XY medium, the Δ*rpfC* strain grew poorly with a maximum OD_600_ of 0.3 and produced very low levels of DSF-family signals (Fig. 2a). Addition of 2.0 g/L sucrose to XY medium (XYS) significantly improved *Xcc* growth to an OD_600_ maximum of 0.8. In XYS medium after 24 h of growth, the Δ*rpfC* strain produced two DSF-family signals BDSF (1.10 μM or 3.7 × 10^3^ μmol/10^9^ cells) and DSF (0.19 μM or 0.63 × 10^−3^ μmol/10^9^ cells) (Fig. 2b). An extremely low level of IDSF (0.005–0.008 μM) and no CDSF were detected in the supernatant of *ΔrpfC* cultured in XYS medium. The addition of starch (medium XYT), glucose (medium XYG) or fructose (medium XYF) to medium XY at 2 g/L had effects similar to those of sucrose on bacterial growth. In media XYT, XYG and XYF, more than 80.0% of the DSF-family signals produced by the Δ*rpfC* strain were BDSF and the DSF levels are relatively stable (Fig. 2a).

### Leucine or valine is required for DSF biosynthesis, and isoleucine is required for the biosynthesis of IDSF

**Figure 3:**
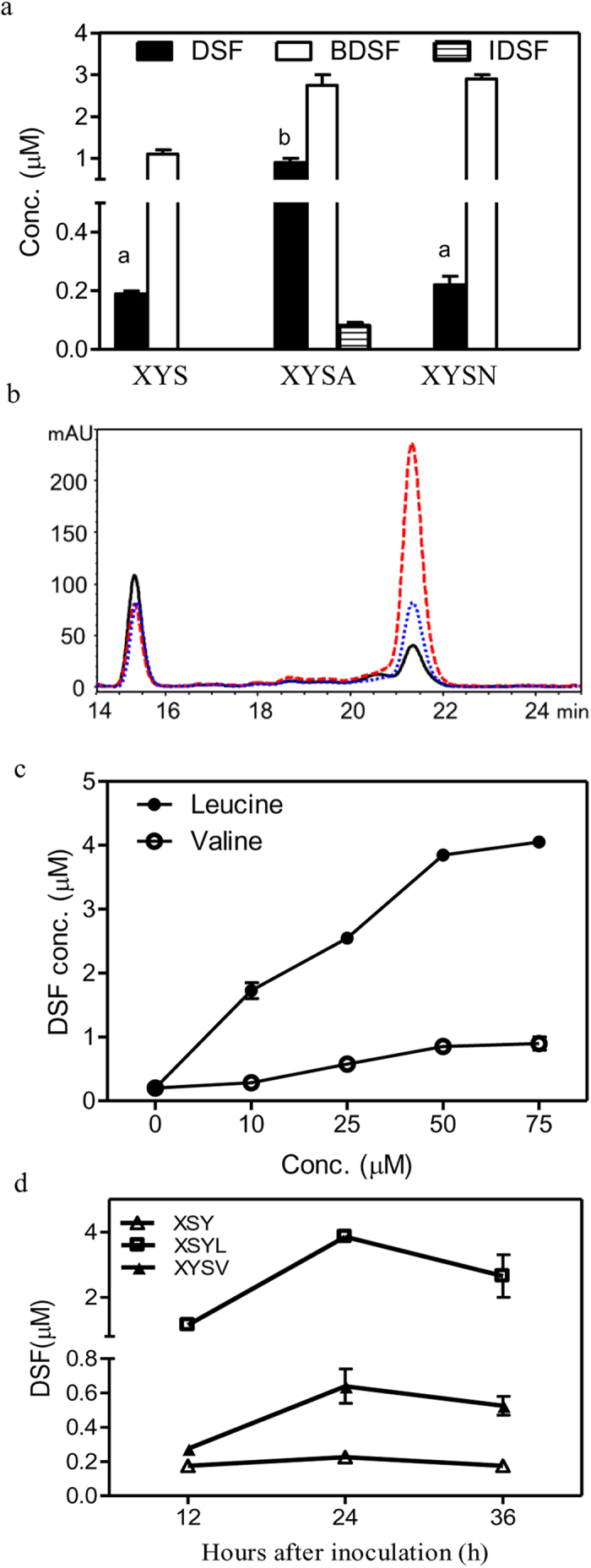
The effects of leucine and valine on DSF biosynthesis. (**a**) Effect of total amino acids and total non-branched-chain amino acids on DSF-family signals production. XYSA: XYS medium supplemented with 100 μM total amino acids; XYSN: XSY medium supplemented with 100 μM non-branched-chain amino acids. (**b**) HPLC analysis of DSF and BDSF production by the Δ*rpfC* strain in media XYS (as indicated in black line), XYSL (red line) and XYSV (blue line) at 24 h after inoculation. XYSL: XYS medium supplemented with 50 μM leucine. XYSV: XYS medium supplemented with 350 μM valine. (**c**) The dose–response curve between leucine or valine concentration and DSF production. (**d**) DSF production time course of the Δ*rpfC* strain in media XYS, XYSL, and XYSV. Data are means ± one standard deviation of three independent assays. Different letters indicate significant differences between treatments (LSD at *P* = 0.05).

To investigate whether amino acids influence the production of DSF-family signals, total amino acids or total amino acids without branched-chain amino acids (BCAAs) at 100 μM were added separately to medium XYS. The resulting media, XYSA and XYSN respectively, supported the growth of the Δ*rpfC* strain to a maximum OD_600_ of 1.2. In XYSA medium, the *ΔrpfC* strain produced three DSF-family signals, DSF (0.9 μM or 2.0 × 10^−3^ μmol/10^9^ cells), BDSF (2.8 μM or 6.2 × 10^−3^ μmol/10^9^ cells) and IDSF (0.08 μM or 0.18 × 10^−3^ μmol/10^9^ cells) (Fig. 3a). However, the *ΔrpfC* strain grown in XSYN medium produced only two DSF-family signals, DSF at a reduced level (0.22 μM or 0.48 × 10^−3^ μmol/10^9^ cells), and BDSF (2.9 μM or 6.4 × 10^−3^ μmol/10^9^ cells) (Fig. 3a). These results suggest that BCAAs are important for both DSF and IDSF biosynthesis.

To further investigate the effects of BCAAs on DSF and IDSF production, leucine, valine and isoleucine were added separately to medium XYS, and DSF-family signal production of the Δ*rpfC* strain at 12 h, 24 h and 36 h was determined. The addition of leucine (50 μM) to medium XYS (XYSL) significantly increased DSF production, but slightly reduced BDSF production (Fig. 3b). A dose–response relation between leucine concentration and DSF production was observed (Fig. 3c). In medium XYS supplemented with 50 μM leucine (XYSL), the *ΔrpfC* strain produced 3.9 μM (13.0 × 10^−3^ μmol/10^9^ cells) DSF after 24 h (Fig. 3d), which was significantly higher than what was observed in XYS medium (0.19 μM or 0.63 × 10^−3^ μmol/10^9^ cells). The addition of valine (10–75 μM) to medium XYS only slightly increased DSF production (Fig. 3c) and had little effect on BDSF production (data not shown). The addition of higher concentrations of valine (200–500 μM) significantly increased DSF production (Fig. 3b). In medium XYS medium supplemented with 350 μM valine (XYSV), the *ΔrpfC* strain produced 0.64 μM (2.1 × 10^−3^ μmol/10^9^ cells) of DSF after 24 h of growth, which is significantly higher than what was observed in XYS medium (0.19 μM or 0.63 × 10^−3^ μmol/10^9^ cells) (Fig. 3d).

**Figure 4:**
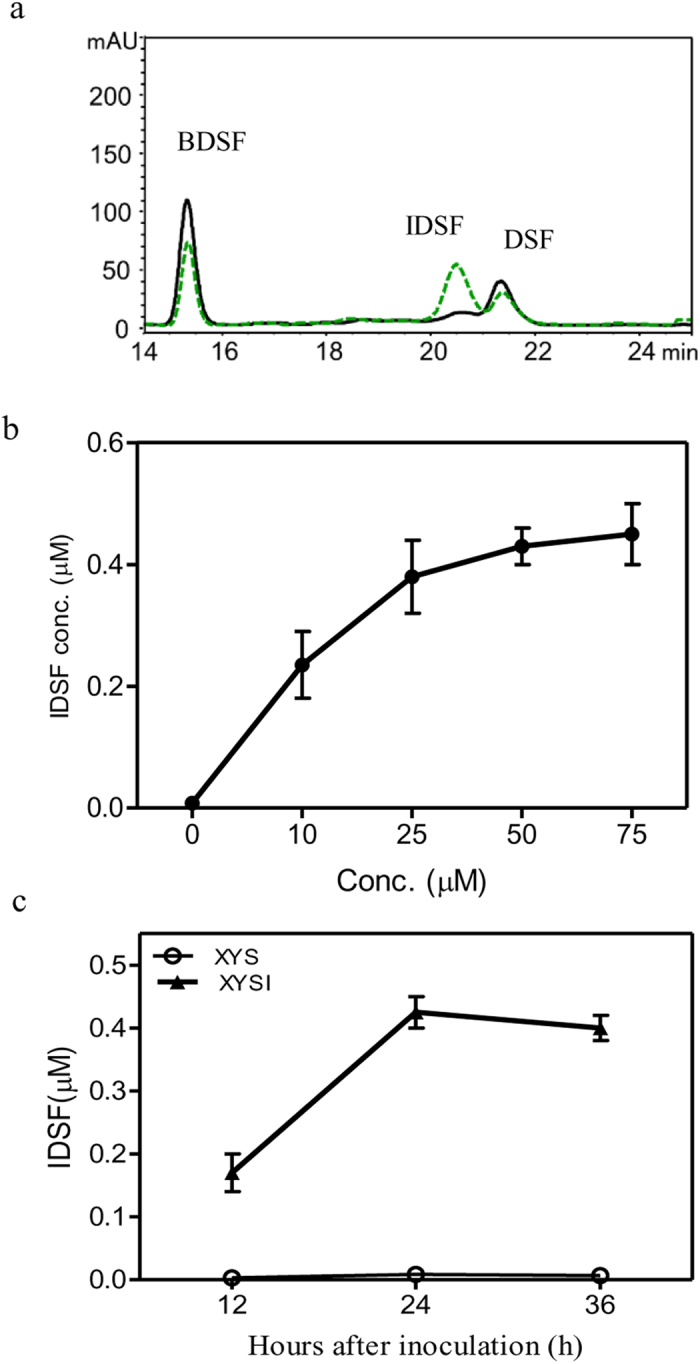
The effects of isoleucine on IDSF biosynthesis. (**a**) HPLC analysis of DSF, BDSF and IDSF production in media XYS and XYSI. XYSI: XYS medium supplemented with 50 μM isoleucine. (**b**) The dose–response curve between isoleucine concentrations (10 to 75 μM) and IDSF production. (**c**) IDSF production time course of the Δ*rpfC* strain in XYSI medium. Data are means ± one standard deviation of three independent assays.

Supplementation of medium XSY with isoleucine (50 μM) led to IDSF production and a slight decrease in DSF and BDSF production (Fig. 4a). IDSF levels increased proportionately with increasing concentrations (10–75 μM) of isoleucine (Fig. 4b). The Δ*rpfC* strain produced 0.42 μM (1.4 × 10^−3^ μmol/10^9^ cells) of IDSF in medium XYS medium supplemented with 50 μM isoleucine (XYSI) after 24 h of growth (Fig. 4c).

### RpfF has both acyl-ACP thioesterase and dehydratase activity, and 3-hydroxydodecanoyl-ACP is the biosynthetic precursor of BDSF

**Figure 5:**
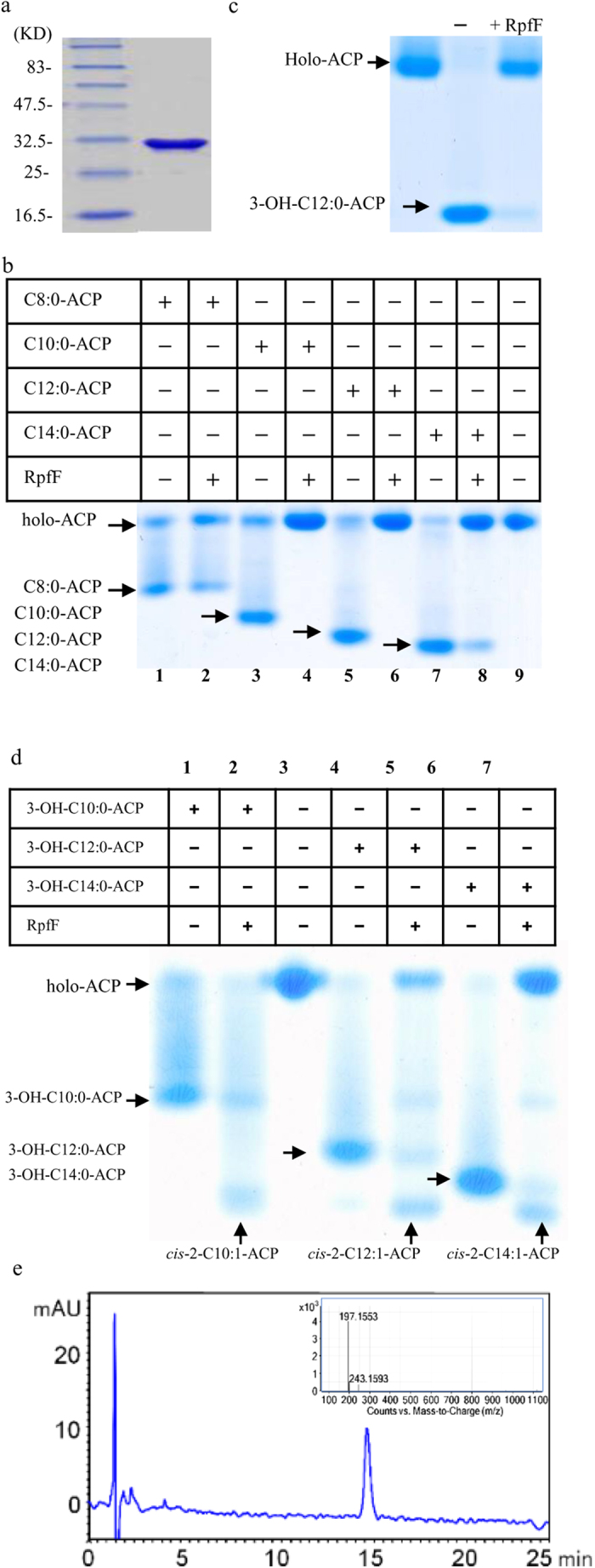
The effect of RpfF on acyl-ACP thioesters. (**a**) SDS-PAGE electrophoresis of purified RpfF protein. (**b**) RpfF reaction with acyl-ACPs, showing that RpfF cleaves acyl-ACP thioester bonds to release holo-ACP (lanes 2, 4, 6, 8); lane 9 contains only holo-ACP as a control. C8:0-ACP, octanoyl-ACP; C10:0-ACP, decanoyl-ACP; C12:0-ACP, dodecanoyl-ACP; C14:0-ACP, tetradencanoyl-ACP. (**c**) RpfF reaction with 3-hydroxydodecanoyl-ACP (3-OH-C12:0-ACP). Acyl-ACPs were first prepared as described in Materials and Methods. (**d**) RpfF reactions with 3-hydroxyacyl-ACPs. The *cis*-2-acyl-ACPs formed are indicated by arrows (lanes 2, 5, 7). Acyl-ACPs were prepared as described in Materials and Methods. (**e**) HPLC analysis of DSF extracts in the reaction mixture containing RpfF and 3-OH-C12:0-ACP (lane 5). LC-MS analysis showed that the active product in HPLC elute is BDSF.

*B. cepacia* BDSF synthase Bcam0581 was shown to have both acyl-ACP thioesterase and dehydratase activity^17^. This finding inspired us to further investigate the enzymatic activity of RpfF in *Xcc*. We tagged RpfF with hexa-histidine and purified it by affinity chromatography (Fig. 5a). To assay the thioesterase activity, RpfF and the acyl-ACP substrates were incubated in a reaction mixture containing 20 mM Tris-HCl and 2 mM β-mercaptoethanol at 37 °C for 30 min. RpfF converted the acyl-ACP substrates into holo-ACP, the unacylated species, indicating the presence of thioesterase activity (Fig. 5b, lanes 2, 4, 6, 8). Among the acyl-ACP substrates tested, decanoyl-ACP (C10:0-ACP) and dodecanoyl-ACP (C12:0-ACP) were found to be optimum for RpfF activity, and almost all the substrates were converted into free fatty acids and holo-ACPs (Fig. 5b, lanes 4 and 6). RpfF could also use 3-hydroxydodecanoyl-ACP (3-OH-C12:0-ACP) as a substrate and effectively cleave acyl-ACP thioester bonds to release holo-ACP (Fig. 5c).

To assay the dehydratase activity, RpfF and 3-OH-acyl-ACP substrates (3-OH-C10:0-ACP, 3-OH-C12:0-ACP, 3-OH-C14:0-ACP) were separately incubated in Tris-HCl buffer (pH 7.5) containing ATP, Mg^2+^ and *Vibrio harveyi* acyl-ACP synthetase (AasS). After incubation at 37 °C for 30 min, new bands corresponding to *cis*-2-C10:1-ACP, *cis*-2-C12:1-ACP, and *cis*-2-C14:1-ACP were observed (Fig. 5d, lanes 2, 5, 7) indicating the presence of dehydratase activity. A compound with an elution time at 15.1 min was detected in the reaction mixture containing RpfF and 3-OH-C12:0-ACP (Fig. 5d, lane 5, and E). Further LC-MS analysis confirmed that it was BDSF (Fig. 5e). In the absence of RpfF, no BDSF was detected in the reaction mixture containing *cis*-2-C12:1-ACP (Fig. 5d, lane 4).

### DSF-family signals are synthesized via the classical FAS elongation cycle

**Figure 6:**
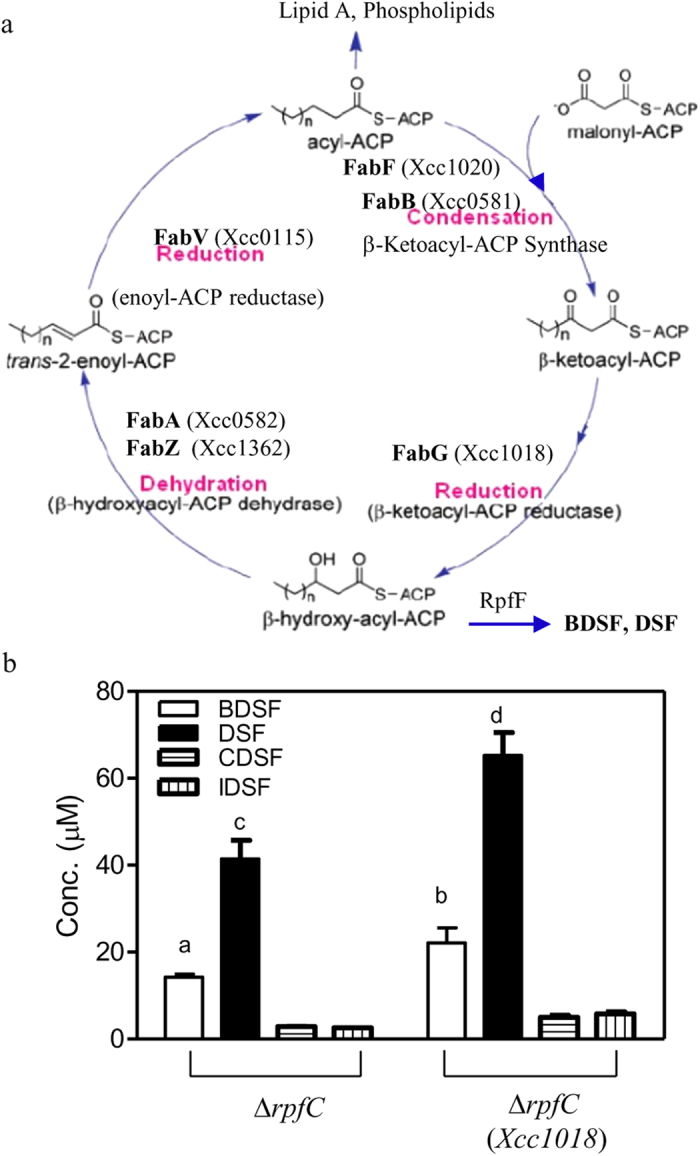
The FabG-encoding gene *Xcc1018* is involved in DSF, BDSF, CDSF and IDSF biosynthesis in *Xcc*. (**a**) The proposed FAS elongation cycle in *Xcc* strain ATCC33913. The enzymes composing the FAS elongation cycle (FabA, FabB, FabF, FabG, FabZ) were proposed based on BLASTP analysis of their counterparts in *E. coli*. (**b**) The effect of overexpression of *Xcc1018* via the expression vector pBBR-1-MCS2 on DSF-family biosynthesis 24 h after inoculation. The data are the means ± one standard deviation of three independent assays. Different letters indicate significant differences between treatments (LSD at *P* = 0.05).

In bacteria, the intermediate 3-OH-acyl ACPs are usually derived from the FAS elongation cycle^17^. *Xcc* contains all of the genes required for the FAS elongation cycle, including *Xcc0582* (FabA), *Xcc0581* (FabB), *Xcc1020* (FabF), *Xcc1018* (FabG), *Xcc0115* (FabV) and *Xcc1362* (FabZ) (Fig. 6a). This led us to further investigate whether DSF-family signals are synthesized via the FAS elongation cycle in *Xcc.* Since the key enzyme β-ketoacyl-ACP reductase (FabG) is directly responsible for the synthesis of 3-OH-acyl ACPs in the FAS elongation cycle (Fig. 6a)^22^, we first attempted to delete the FabG-encoding gene *Xcc1018*. However, these experiments were not successful probably due to the fact that *Xcc1018* is an essential gene. As an alternative approach, we overexpressed *Xcc1018* in the Δ*rpfC* strain, and BDSF and DSF levels were examined. The results showed that overexpression of *Xcc1018* in strain Δ*rpfC* had no significant effect on *Xcc* growth in NA medium, but led to a significant increase in the production of DSF, BDSF, CDSF and IDSF at 24 h (Fig. 6b).

**Figure 7:**
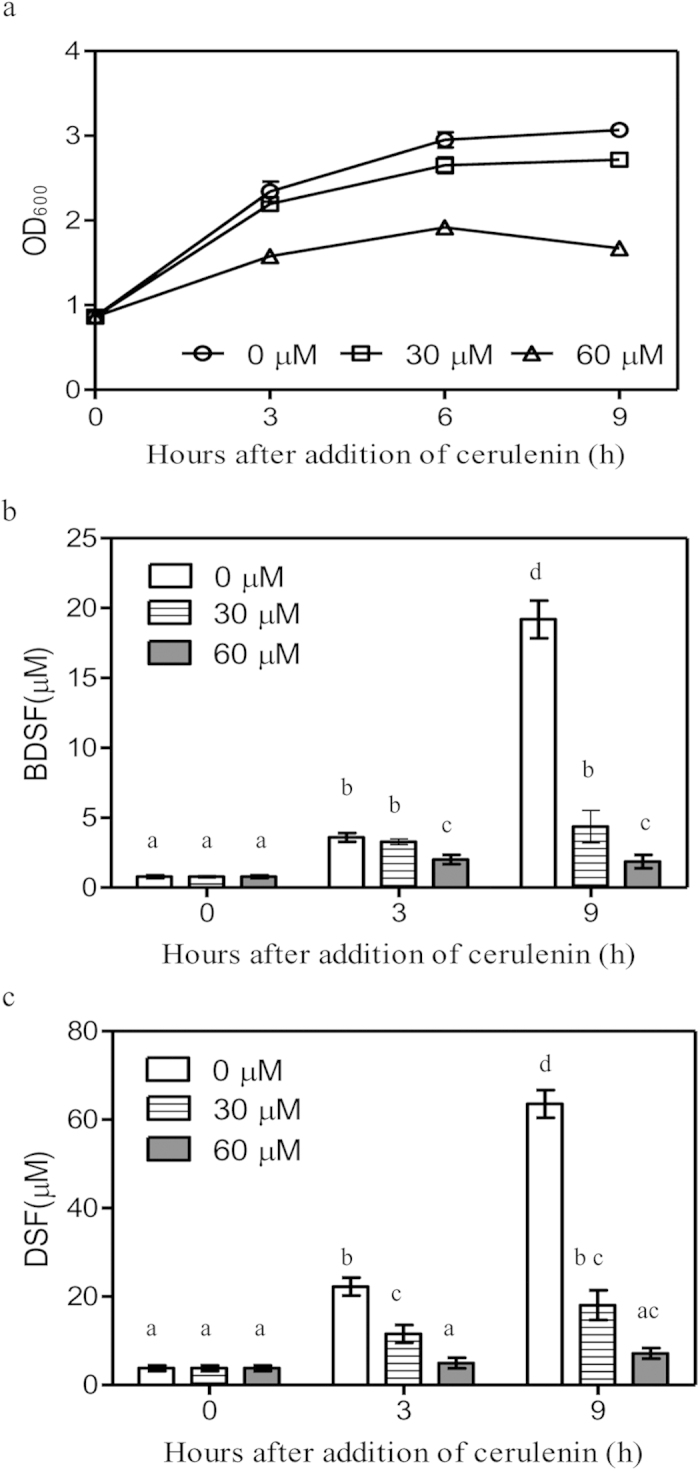
The effects of cerulenin on bacterial growth and the production of BDSF and DSF in *Xcc*. Bacterial growth (**a**), supernatant BDSF levels (**b**) and supernatant DSF levels (**c**) of the *ΔrpfC* strain after the addition of cerulenin to NA liquid medium. Cerulenin (3 mg/ml) was added to the cultures at OD_600_ of 1.0. The data are the means ± one standard deviation of three independent assays. Different letters indicate significant differences between treatments (LSD at *P* = 0.05).

To further verify the roles of FAS elongation cycle, cerulenin, an antibiotic that binds in equimolar ratio to long chain 3-keto-acyl-ACP synthases (FabF and FabB)^35^,^36^, was used to block fatty acid synthesis. The minimum inhibitory concentration of cerulenin on *Xcc* growth in liquid NA medium was 30 μg/ml. The OD_600_ of the cell culture treated with cerulenin (30 μg/ml) for 9 h was 2.7, which was a little lower than the OD_600_ of 3.1 of the untreated cell culture (Fig. 7a). DSF level of the cerulenin-treated culture was 11.5 μM (5.5 × 10^−3^ μmol/10^9^ cells), and this was about one fifth of 63.5 μM (25.0 × 10^−3^ μmol/10^9^ cells) which was found in untreated cell cultures (Fig. 7b). Similarly, the BDSF level of the cerulenin-treated culture was 4.3 μM (2.0 × 10^−3^ μmol/10^9^ cells), and this was about one-fourth of the levels found in the untreated cell cultures (19.1 μM or 7.6 × 10^−3^ μmol/10^9^ cells) (Fig. 7c). Neither CDSF nor IDSF were detected in the supernatant of cerulenin-treated *Xcc* culture.

## Discussion

The DSF-based quorum sensing system has emerged as another widely conserved cell-cell communication mechanism in Gram-negative bacteria. Previous studies showed that *Xoo* and *Burkholderia* sp. produce DSF, BDSF and/or CDSF, and that the amounts and relative proportions of these signals are influenced by culture medium composition^6^,^7^. The present study showed that this is also the case with *Xcc,* with the addition of a fourth signal, IDSF (Fig. 1c). IDSF is a new DSF-family signal (*cis*-10-methyl-2-dodecenoic acid), which is otherwise identical to DSF except for the methyl group at position C10. IDSF is also similar to DSF in its effects on aggregate dispersal, EPS production and protease activity (Supplementary Fig. S4), suggesting that it is a functional DSF-family signal. However, the levels of CDSF and IDSF are much lower than those of DSF and BDSF in the supernatant of *Xcc* cell culture (Fig. 1), suggesting that their roles in *Xcc* quorum sensing might be limited.

By using a range of defined media, the present study shows the metabolic origins of the DSF-family signals in *Xcc*. First, *Xcc* mainly produces BDSF in the presence of carbohydrates (Fig. 2a). Second, leucine is required for DSF biosynthesis (Fig. 3). Third, isoleucine is required for IDSF biosynthesis (Fig. 4). Valine is one carbon less than leucine; however, the addition of high concentration of valine (350 μM) also induced DSF biosynthesis at lower level (Fig. 3). This is probably due to the fact that valine is converted into α-ketoisovalerate (KIV), which can be further used for leucine biosynthesis^36^. In general, these data are consistent with the findings in some Gram-positive bacteria that branched-chain fatty acid are synthesized from branched-chain amino acids^37^. These findings explain why *Xcc* and *Xoo* produce multiple DSF-family signals in rich YEB or NA medium. Both media contain sucrose and a high concentration of tryptone, peptone or yeast extract, which provides a rich source of amino acids including branched-chain amino acids. Nevertheless, this study does not rule out the possibility that *Xanthomonas* may use other nutrient sources such as non-branched amino acids, short chain carboxylic acids and lipids for DSF-family signal biosynthesis. The results in Fig. 3a show that the addition of non-BCAAs to XYS medium (XYSN) increased BDSF production, suggesting that some of them might be involved in BDSF biosynthesis. Further study is needed to investigate the roles of the non-BCAAs, in particular, those present in plant xylem fluids^38^,^39^.

The present study confirms that *Xcc* RpfF has the same acyl-ACP thioesterase and dehydratase activities as Bcamo581 in *Burkholderia*^17^, and is active on acyl-ACPs ranging from 8 to 14 carbons in length (Fig. 5). RpfF is essential for the production of DSF, BDSF, CDSF and IDSF, suggesting that RpfF could accommodate and use multiple types of acyl-ACP precursors for the synthesis of different DSF-family signals. Considering that C12:0-ACP is an optimum substrate for RpfF (Fig. 5) and that DSF, BDSF, CDSF and IDSF are all 12 carbon chains, it seems likely that RpfF specifically recognizes C12-acyl-ACP substrates for DSF-family signal production, although the basis for this is not known. The 3-D structural analysis showed that RpfF contains a hydrophobic pocket, which is probably a DSF precursor docking site^16^. It is possible that RpfF selectively binds only those substrates that are easily accommodated by the pocket. Recently, Bi *et al.*^19^ showed that RpfB is a fatty acyl-CoA ligase required to counteract the thioesterase activity of RpfF. In our observations with *Xcc* (data not shown), deletion of *rpfB* significantly increases the levels of all four DSF-family signals in NA medium, further confirming the role of RpfB as described by Bi *et al.*^19^.

**Figure 8:**
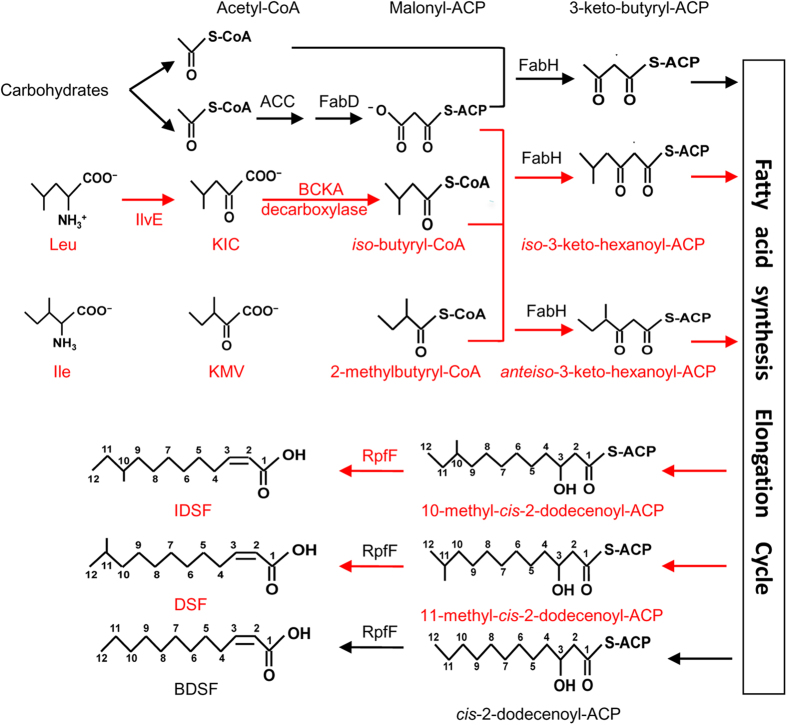
A schematic model for the general biosynthetic pathway of DSF, BDSF and IDSF in *Xanthomonas*. BCAA: branched-chain amino acids; BCKA: branched-chain α-ketoacids. ACC: acetyl-CoA carboxylase. KIC: 2-keto-isocaproic acid. KMV: 2-keto-β-methylvaleric acid.

The present study shows that DSF-family signals are synthesized via the essential FAS elongation cycle (Figs 6 and 7). On the basis of previous and present findings, a general biosynthetic pathway for DSF-family signals is proposed (Fig. 8). In the presence of carbohydrates, acetyl-CoA is produced via aerobic cellular respiration and is further converted into malonyl-CoA by acetyl-CoA carboxylase (ACC). Malonyl-ACP is synthesized from ACP and malonyl-CoA by FabD (*Xcc1016*), which is then condensed with acetyl-CoA by FabH (*Xcc1017*) to form 3-keto-butyl-ACP for the initial step of the FAS elongation cycle. The intermediate 3-hydroxydodecanoyl-ACP is formed via the FAS elongation cycle. RpfF catalyzes the synthesis of BDSF by using 3-hydroxydodecanoyl-ACP as a substrate. In the presence of carbohydrates, leucine and isoleucine (such as in the rich media YEB or NA), the branched-chain amino acid aminotransferase IlvE encoded by Xcc0850 catalyzes the deamination of leucine and isoleucine to form 2-keto-isocaproic acid (KIC) and 2-keto-β-methylvaleric acid (KMV) respectively, which are then further acted upon by α-ketoacid dehydrogenase (BCKA) to form *iso-*butyryl-CoA and 2-methylbutyryl-CoA respectively. Malonyl-ACP is then condensed with these acyl-CoAs to form 3-keto-butyl-ACP, *iso*-3-keto-hexanoyl-ACP and *anteiso*-3-keto-hexanoyl-ACP for the initial step of the FAS cycle. The intermediates 3-hydroxydodecanoyl-ACP, 11-methyl-*cis*-2-dodecenoyl-ACP and 10-methyl-*cis*-2-dodecenoyl-ACP are formed via the FAS elongation cycle. These acyl-ACP intermediates compete for binding to RpfF. The relative proportion of DSF, BDSF and IDSF formed is determined by the relative concentrations of acyl-ACP intermediates and their affinities for RpfF. Since 11-methyl-3-hydroxydodecanoyl-ACP is more hydrophobic than 3-hydroxydodecanoyl-ACP, and the DSF biosynthetic pocket in RpfF is highly hydrophobic^16^, 11-methyl-3-hydroxydodecanoyl-ACP gains easier access to the biosynthetic pocket than 3-hydroxydodecanoyl-ACP. This might explain why more DSF, rather than BDSF, is produced in the rich media YEB or LB and why BDSF production was reduced in the leucine-rich XYSL medium or the isoleucine-rich XYSV medium (Figs 3 and 4). Further insights into this proposed pathway will be gained from the experimental analysis of FabD, FabH, ACC, and BCKA in *Xcc*.

## Methods

### Bacterial strains and growth conditions

*Xcc* wild type strains XC1, 8004 and their derivatives were described previously^40^. *Xcc* strains were routinely grown at 30 °C in the media shown in Supplementary Table 1 with 25 μg ml^−1^ of rifampicin. For media preparation (Supplementary Table 2), tryptone, peptone and yeast extract were purchased from Sangon Biotech (China). *Escherichia coli* strains were grown at 37 °C in LB medium. Bacterial growth was determined by measuring optical density at 600 nm.

### Extraction and purification of DSF-family signals from bacterial culture supernatants

The methods for DSF extraction were described previously by He *et al.*^6^. The ethyl acetate fractions were collected, and the solvent was removed by rotary evaporation at 40 °C to dryness. The crude extract was subjected to a 0.45 μm Minisart filter unit and was then condensed to 0.5 ml. Three microliters of the extract was subjected to HPLC using a C18 reverse-phase column (4.6 × 150 mm, Agilent), and eluted with water in methanol (23:77, v/v, 0.1% formic acid) at a flow rate of 1 ml/min in a Agilent Technologies 1260 Infinity system with DAD G1315D VL detector.

### Bioassay and quantitative analysis of DSF-family signals

DSF bioassays were performed as described previously^5^. DSF, BDSF and IDSF levels in the culture supernatant were quantified using peak area (A) in HPLC elute according to the following formula: DSF (μM) = 1.32A-50.24, BDSF (μM) = 0.71A-10.64, IDSF (μM) = 1.27A-49.36. The formula was derived from a dose–peak area plot in HPLC elute using various dilutions of synthetic DSF and BDSF signals with a correlation coefficient (R^2^) of 0.999 and 0.998, respectively. To show the efficiency of DSF production (the amount of DSF produced per cell) in different media, DSF concentration (μM) was normalized into DSF yield (μmol/10^9^ cells) based on cell number (N). The cell number N (cells/ml) was quantified using OD_600_ according to the following formula: N = 10^7^ × 5.6492e^1.6916OD600^. The formula was derived from an OD_600_ value-cell number plot with a correlation coefficient (R^2^) of 0.9918.

### Liquid Chromatography-Mass Spectrometry and Nuclear Magnetic Resonance analysis

Five microliters of the collected samples were applied to an Ultra Performance Liquid Chromatographic system (UPLC, Agilent 1290 Infinity) on a Zorbax XDB C18 reverse phase column (4.6 × 150 mm, temperature-controlled at 30 °C), and eluted with methanol-water (80:20, v/v) at a flow rate of 0.4 ml/min in a diode array detector (Agilent G4212A). ^1^H, ^13^C, ^1^H-^1^H COSY, and heteronuclear single quantum coherence (HSQC) nuclear magnetic resonance (NMR) spectra in CH_3_DO solution were obtained using a Bruker Avance III 600 MHz.

### Gene deletion and overexpression analysis

The in-frame deletion mutant of *Xcc* strain XC1 was generated following the method previously described^40^ using the primers listed in Supplementary Table 1. For complementation analysis, the coding region of the target gene was amplified by PCR using the primers listed in Supplementary Table 1, and was then placed under the control of the *lac* promoter in pBBR-1-MCS. The resulting construct was then transferred to *Xcc* strains via tri-parental mating.

### Cell aggregation assay and quantitative determination of extracellular protease activity and EPS production

Cell aggregation assay and quantitative analysis of extracellular polysaccharide in the supernatants of *Xcc* cell cultures were previously described by He *et al.*^40^. The extracellular protease activity in culture supernatants of *Xcc* strains was analyzed according to the method described previously by Swift *et al.*^41^. Each experiment was repeated at least twice, and the data shown are the average of three replicates with standard deviation.

### Protein expression and purification

RpfF expression was performed as described previously by He *et al.*^14^. The supernatant containing His-tagged RpfF was then incubated with Ni^2+^ resin pre-equilibrated with column buffer (25 mM Tris buffer pH 8.0 and 500 mM NaCl).

### Acyl-ACP preparations

The acyl-ACPs used in this study were synthesized as described previously^17^. Briefly, 1 ml of reaction mixture consisting of 20 μM holo-ACP, 200 μM fatty acid, 170 μM *V. harveyi* AasS in a buffer containing 100 mM Tris-HCl (pH7.8), 10 mM MgCl_2_, 1 mM DTT and 10 mM ATP was prepared. The reaction mixtures were incubated at 37 °C for 4 h and then stopped by the addition of two volumes of acetone. Protein was precipitated at −20 °C overnight and then pelleted at 20,000 × *g* for 30 min at 4 °C. After washing twice with 3 volumes of acetone, the pellets were air-dried and resuspended in 20 mM Tris-HCl (pH7.5).

### Assay of RpfF thioesterase and dehydratase activity

The enzymatic activity assay of RpfF was performed as described previously^17^. Briefly, a reaction mixture containing 0.1 M Tris-HCl (pH7.5), 2 mM β-mercaptoethanol, 20 μM acyl-ACP and 0.2 μg of a purified His-tagged enzyme was prepared in a final volume of 30 μl. The reaction mixtures were incubated at 37 °C for 30 min, and the reaction products were then resolved by conformationally sensitive gel electrophoresis on 18% polyacrylamide gels containing a urea concentration optimized for the separation. The gels were stained with Coomassie Brilliant Blue R250.

### Cerulenin treatment of *Xcc*

Cerulenin was purchased from Enzo Life Sciences and was dissolved in ethanol for a stock solution (3 mg ml^−1^). *Xcc* strains were grown overnight in NA liquid medium at 37 °C. Cell pellets were then collected by centrifugation at 6,000 × g for 15 min. After washing twice using fresh NA liquid medium, pellets were re-suspended in NA medium to an OD_600_ of 1.0. Cerulenin was added to the culture at a final concentration ranging from 20 to 60 μg ml^−1^. After further growth for 3 h and 6 h, 10 ml of cell culture were collected for DSF and BDSF extraction.

### Statistical analysis

Analysis of variance for experimental datasets was performed using JMP software version 5.0 (SAS Institute Inc., Cary, NC). Significant effects of treatment were determined by the F test (P = 0.05). When a significant F value was obtained, the separation of means was accomplished by Fisher’s protected LSD (least significant difference) at P = 0.05.

## Additional Information

**How to cite this article**: Zhou, L. *et al.* The Multiple DSF-family QS Signals are Synthesized from Carbohydrate and Branched-chain Amino Acids via the FAS Elongation Cycle. *Sci. Rep.* **5**, 13294; doi: 10.1038/srep13294 (2015).

## Supplementary Material

Supplementary Information
