# Supplementary material for: The Multiple DSF-family QS Signals are Synthesized from Carbohydrate and Branched-chain Amino Acids via the FAS Elongation Cycle
Source: Sci Rep. 2015 Aug 20;5:13294. doi: 10.1038/srep13294 (PMC4542539; doi:10.1038/srep13294)
Supplement: Supplementary Information [file srep13294-s1.pdf]

Supplementary information

Title: The Multiple QS DSF-family Signals are Synthesized from Carbohydrate and Branched-chain Amino Acids via the FAS Elongation Cycle

Lian Zhou[1], Yonghong Yu[2]， Xiping Chen[3], Abdelgader Abdeen Diab [1]， Lifang Ruan, Jin He, Haihong Wang[2,*], Ya-Wen He[1,*]

Contents

Supplementary Table S1

Supplementary Table S2

Supplementary Figure S1

Supplementary Figure S2

Supplementary Figure S3

Supplementary Figure S4

Supplementary Table S1. Bacterial strains, plasmids and primers used in this study

	Relevant characteristics	Reference/ source
Strains		
DH5α	*E. coli* F– Φ80*lac*ZΔM15 Δ(*lac*ZYA-argF) U169 *recA*1 *endA*1 *hsdR*17 (rK–, mK+) *phoA supE*44 λ– thi-1 *gyr*A96 *relA*1	Lab stock
BL21	*E. coli* B F- *dcm ompT hsdS*(r_B- m_B-) gal [*malB*$^+$]$_{K-12}$(λ^S)	Lab stock
XC1	*Xcc* wild-type strain	Lab stock
Δ*rpfF*	The *rpfF* in-frame deletion mutant of strain XC1	1
Δ*rpfC*	The *rpfC* in-frame deletion mutant of strain XC1	2
FE58	The ΔrpfF strain containing pLAFR3-P*engXCA-gusA*, in which the coding region of *E. coli* β-glucuronidase was placed under the control of DSF-inducible promoter P*engXCA*, Tc^R.	3
Plasmids		
pLAFR3	A wide-host-range cosmid cloning vector, Tc^R	4
pK18mobsacB	A mobilizable vector, allows for selection of double crossover in *Xcc*, Kan^R	1
pBBR-1-MCS2	5	5
pET-14b-*rpfF*	pET-14b containing *rpfF*, Amp^R	2
Primers		
Xcc1018 overexpression	1018FOR: CCCaagcttATGAGCAAGCCATTGCAGGGCGAG<br>1018REV: CGggatccCGATGGCCGCGTTGGATAGTGTAG	This study

References:

1. He, Y.W. *et al*. Genome scale analysis of diffusible signal factor regulon in *Xanthomonas campestris* pv. *campestris*: identification of novel cell-cell communication-dependent genes and functions. *Mol Microbiol* **59(2)**, 610-622 (2006).
2. He, Y.W. *et al*. Dual signaling functions of the hybrid sensor kinase RpfC of *Xanthomonas campestr*is involve either phosphorelay or receiver domain-protein interaction. *J Biol Chem* **281(44)**, 33414-33421 (2006).
3. Wang, L.H. *et al.* A bacterial cell-cell communication signal with cross-kingdom structural analogues. *Mol Microbiol* **51(3)**, 903-912 (2004).
4. Staskawicz, B., Dahlbeck, D., Keen, N. & Napoli, C. Molecular characterization of cloned avirulence genes from race 0 and race 1 of *Pseudomonas syringae* pv. *glycinea*. *J Bacteriol* **169(12)**, 5789-5794 (1987).
5. Kovach, M.E. *et al.* Four new derivatives of the broad-host-range cloning vector pBBR1MCS, carrying different antibiotic-resistance cassettes. *Gene* **166(1)**, 175-176 (1995).

Supplementary Table S2. Media used in this study

Media	Composition	Reference or source
LB	10 g L^{-1} tryptone, 5 g L^{-1} yeast extract, 10 g L^{-1} NaCl, pH 7.0	6
YEB	5 g L^{-1} yeast extract, 10 g L^{-1} tryptone, 5 g L^{-1} sodium chloride, 5 g L^{-1} sucrose, 0.5 g L^{-1} MgSO4 , pH 7.0	7
NA	5 g L^{-1} peptone, 3 g L^{-1} beef extract, 10 g L^{-1} sucrose, 1 g L^{-1} yeast extract	8
NYG	5 g L^{-1} peptone, 3 g L^{-1} yeast extract, 20 g L^{-1} glycerol	9
XOLN	K_2HPO_4 0.7 g L^{-1}, KH_2PO_4 0.2 g L^{-1}, $(NH_4)_2SO_4$ 1 g L^{-1}, $MgCl_2$ 0.1 g L^{-1}, $FeSO_4$ 0.01 g L^{-1}, $MnCl_2$ 0.001g L^{-1}, 0.6 g L^{-1} tryptone, 0.6 g L^{-1} yeast extract, pH 7.0	10
XY	XOLN salts, 0.2 g L^{-1} yeast extract, pH 7.0	This study
XYS	XOLN salts, 0.2 g L^{-1} yeast extract, and 2 g L^{-1} sucrose, pH 7.0	This study
XYT	XOLN salts, 0.2 g L^{-1} yeast extract, and 2 g L^{-1} starch, pH 7.0	This study
XYG	XOLN salts, 0.2 g L^{-1} yeast extract, and 2 g L^{-1} glucose, pH 7.0	This study
XYF	XOLN salts, 0.2 g L^{-1} yeast extract, and 2 g L^{-1} fructose, pH 7.0	This study
XYSA	XYS medium supplemented with 100 µM total amino acids and 2 g L^{-1} sucrose, pH 7.0	This study
XYSN	XYS medium supplemented with 100 µM total amino acids except for leucine, isoleucine and valine, and 2 g L^{-1} sucrose, pH 7.0	This study
XYSL	XY Smedium supplemented with 50 µM leucine and 2 g L^{-1} sucrose, pH 7.0	This study
XYSV	XYS medium supplemented with 350 µM valine and 2 g L^{-1} sucrose, pH 7.0	This study
XYSI	XY Smedium supplemented with 50 µM isoleucine and 2 g L^{-1} sucrose, pH 7.0	This study

References:

6. Bertani, G. Studies on lysogenesis. I. The mode of phage liberation by lysogenic *Escherichia coli*. *J Bacteriol* **62**, 293-300 (1951).
7. Zhang, H.B., Wang, L.H. & Zhang, L.H. Genetic control of quorum-sensing signal turnover in *Agrobacterium tumefaciens*. *Proc Natl Acad Sci U S A* **99(7)**, 4638–4643 (2002).
8. Guo, W. *et al*. Identification of seven *Xanthomonas oryzae* pv. *oryzicola* genes potentially involved in pathogenesis in rice. *Microbiology* **158**, 505–518 (2012).
9. Turner, P., Barber, C. & Daniels, M. Behavior of the transposons Tn*5* and Tn*7* in *Xanthomonas campestris* pv. *campestris*. *Mol Gen Genet* **195**, 101-107 (1984).
10. Fu, J.F. & Tseng, Y.H. Construction of lactose-utilizing *Xanthomonas campestris* and production of xanthan gum from whey. *Appl Environ Microbiol***. 56(4)**, 919–923 (1990).

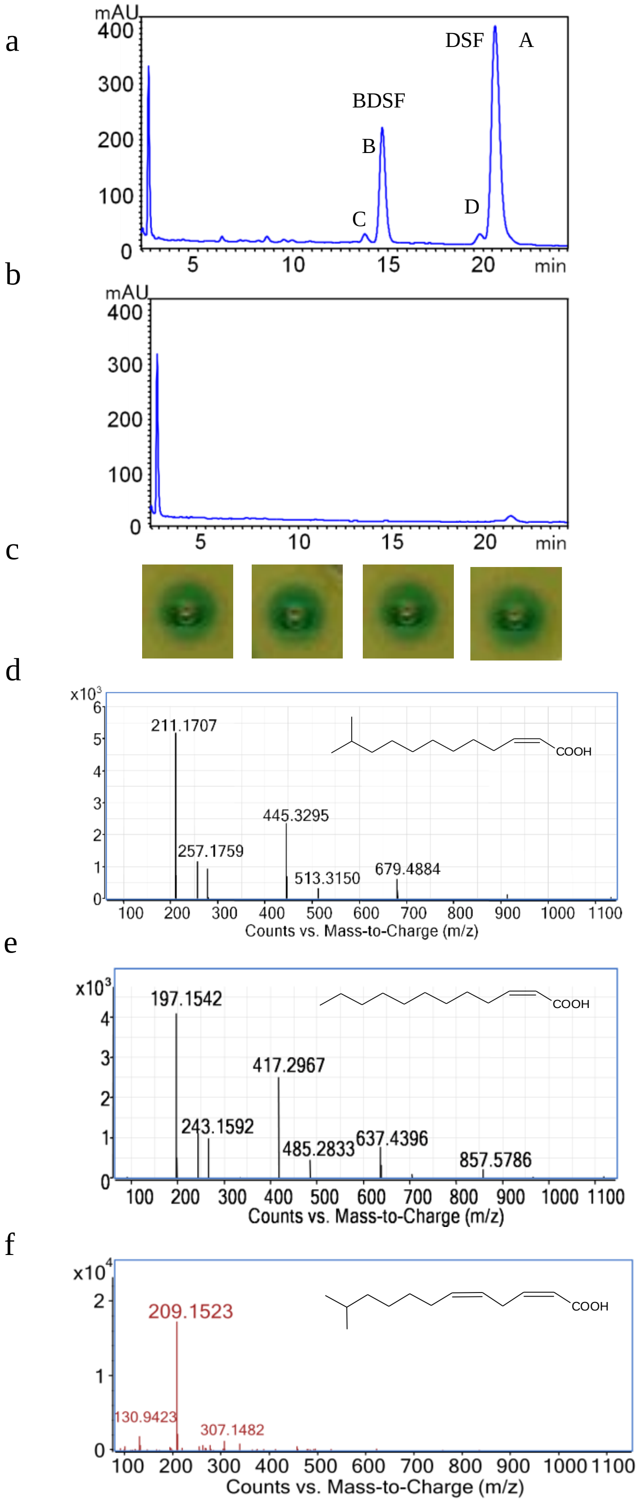

Supplementary Figure S1. *Xcc* produces four DSF-family signals via RpfF. (a) Four DSF-like signals were detected in the supernatant of the Δ*rpfC* strain in LB medium. (b) No DSF-family signals were detected in the supernatant of the Δ*rpfF*Δ*rpfC* strain. (c) Bioassay of compounds A, B, C, D. (d) LC-MS analysis to verify the identity of DSF in peak A (e) LC-MS analysis to verify the identity of BDSF in peak B. (f) LC-MS analysis to verify the identity of CDSF in peak C. The procedures for BDSF and DSF extraction, purification and HPLC analysis was described in Materials and Methods.

a

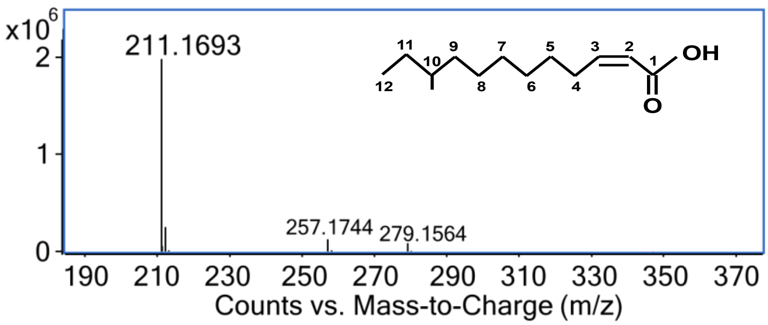

b

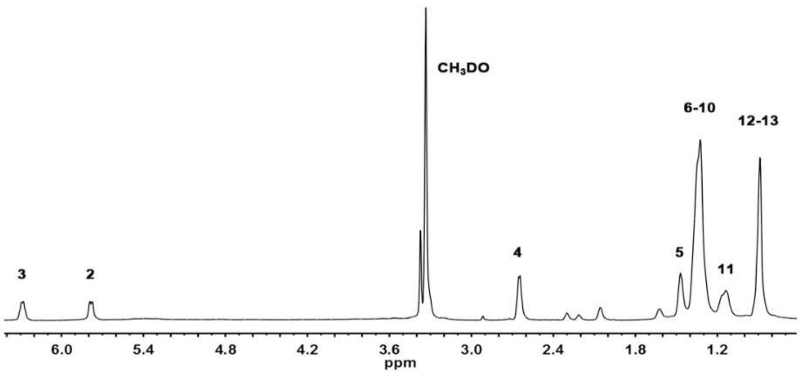

c

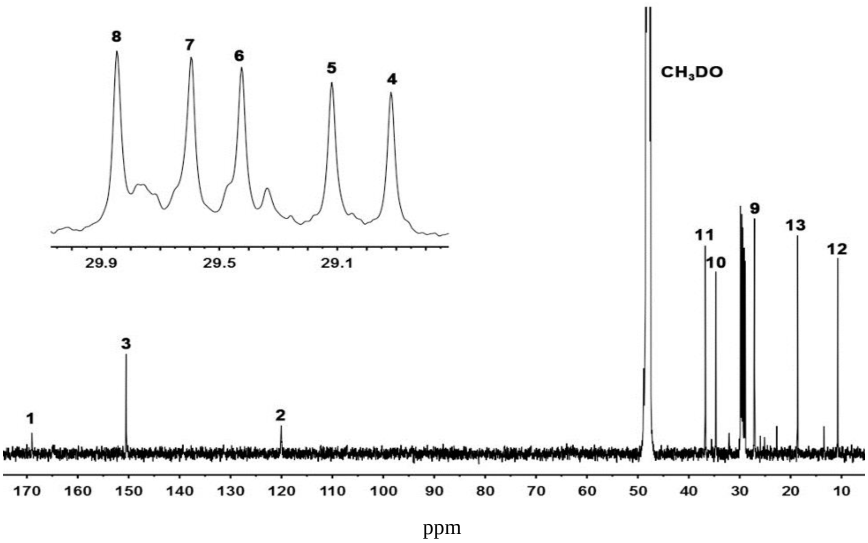

Supplementary Figure S2. IDSF is a novel DSF-family signal. (a) LC-MS analysis of IDSF showing a molecular weight of 212.1693 dalton. (b) The ^{1}H NMR spectra of IDSF. (c) The ^{13}C NMR spectra of IDSF. The NMR analyses were conducted on a Bruker Avance III 600 MHz.

a

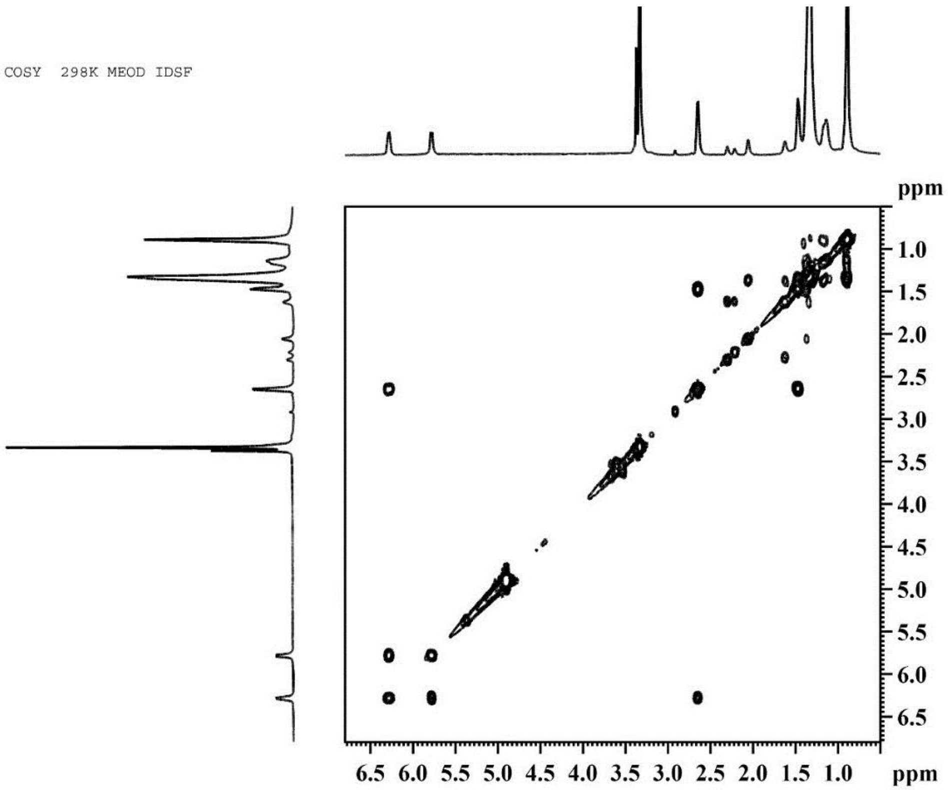

b

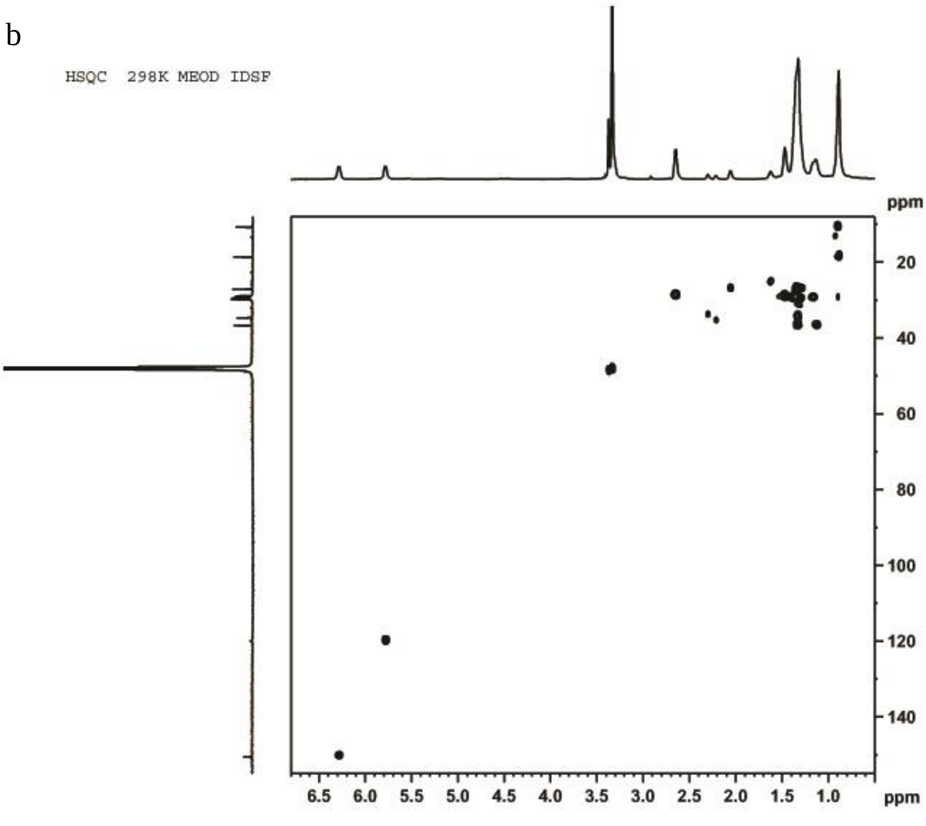

Supplementary Figure S3. IDSF is a novel DSF-family signal. (a) COSY analysis; (b) HSQC analysis. The NMR analyses were conducted on a Bruker Avance III 600 MHz.

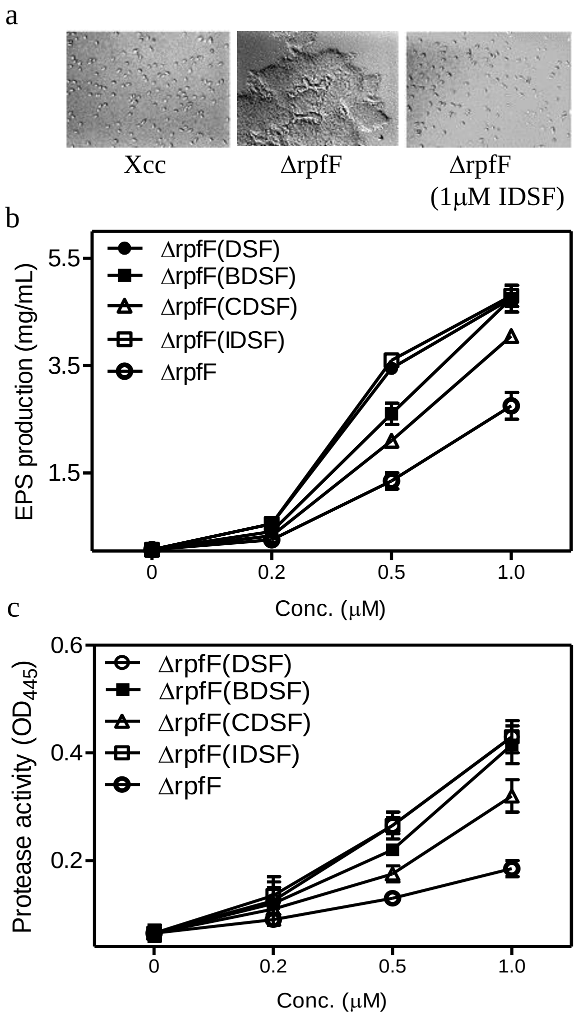

Supplementary Figure S4. The effects of DSF-family signals on biofilm dispersion and the induction of extracellular polysaccharide and protease activity. (a) The effect of IDSF (1 μM) on biofilm dispersion in the *rpfF* mutant of *Xcc* strain 8004 in YEB medium. (b) The effects of DSF, BDSF, CDSF and IDSF (0.2 – 1.0 μM) on EPS production of the *rpfF* mutant of *Xcc* strain XC1. (c) The effects of DSF, BDSF, CDSF and IDSF (0.2 – 1.0 μM) on protease activity of the *rpfF* mutant of *Xcc* strain XC1. Data are means ± one standard deviation of three independent assays.
